# EMI radiation of power transmission lines in Malaysia

**DOI:** 10.12688/f1000research.73067.1

**Published:** 2021-11-10

**Authors:** Azhan Fikry, Siow Chun Lim, Mohd Zainal Abidin Ab Kadir

**Affiliations:** 1 Faculty of Engineering, Multimedia University, Cyberjaya, Selangor, 63100, Malaysia; 2 Electrical & Electronic Engineering, University Putra Malaysia, Serdang, Selangor, 43400, Malaysia

**Keywords:** EMI radiation, power transmission lines, EMF, ELF, public exposure limit, Right-of-Way, ICNIRP

## Abstract

**Background:** There has been rising concern amongst the public regarding their home's proximity to high tension power transmission lines. The primary cause of fear is the impact of the electromagnetic interference (EMI) radiation on the nearby occupants' health. Despite the presence of national permissible limits of EMI radiation, there is still lack of information with regards to the EMI radiation of the types of power lines configuration in Malaysia.

**Methods:** The electric and magnetic fields of several selected power transmission lines were simulated using the EMFACDC software program from the recommendation ITU-T K.90. Five types of power transmission lines available in Malaysia are considered.

**Results:** It was found that the simulated electric and magnetic field levels at all the power lines' right of way (ROW) boundary complies with the prescribed exposure limit. However, the electromagnetic fields (EMF) level increases significantly as the separation distance is reduced from 30m. For a more conservative approach, the ROW can be set at 30m across all transmission voltage level and corridor area condition.

**Conclusion:** It can be concluded that Malaysia's power transmission lines are within the prescribed exposure limits. To further minimize the electric and magnetic field level, it is recommended that the residential building should be built at least 30 meters away from the power transmission lines, especially for the 275kV double circuit, 275/132kV quadruple circuit, and 500kV double circuit lines.

## Introduction

The public is getting increasingly concerned about the potential biological and health effects of power transmission lines considering the risk of exposure to electromagnetic interference (EMI) radiation. Biological effects are noticeable responses to a stimulus or an environmental change. ^1^ At an extreme level, electromagnetic fields (EMF) will affect humans' health, such as micro shocks and induced currents in the body. ^2^ Power transmission lines produce electric and magnetic fields at an extremely low frequency. These transmission lines were sometimes located in close proximity to residential areas, which could potentially increase the exposure level of electromagnetic radiation. Although national and international guidelines have established exposure limits to protect the public against high-level EMF that might be harmful, there are still rising concerns among the public on whether their residency is affected by such radiation, especially to those living near power transmission lines. ^3^

**Figure 1.**
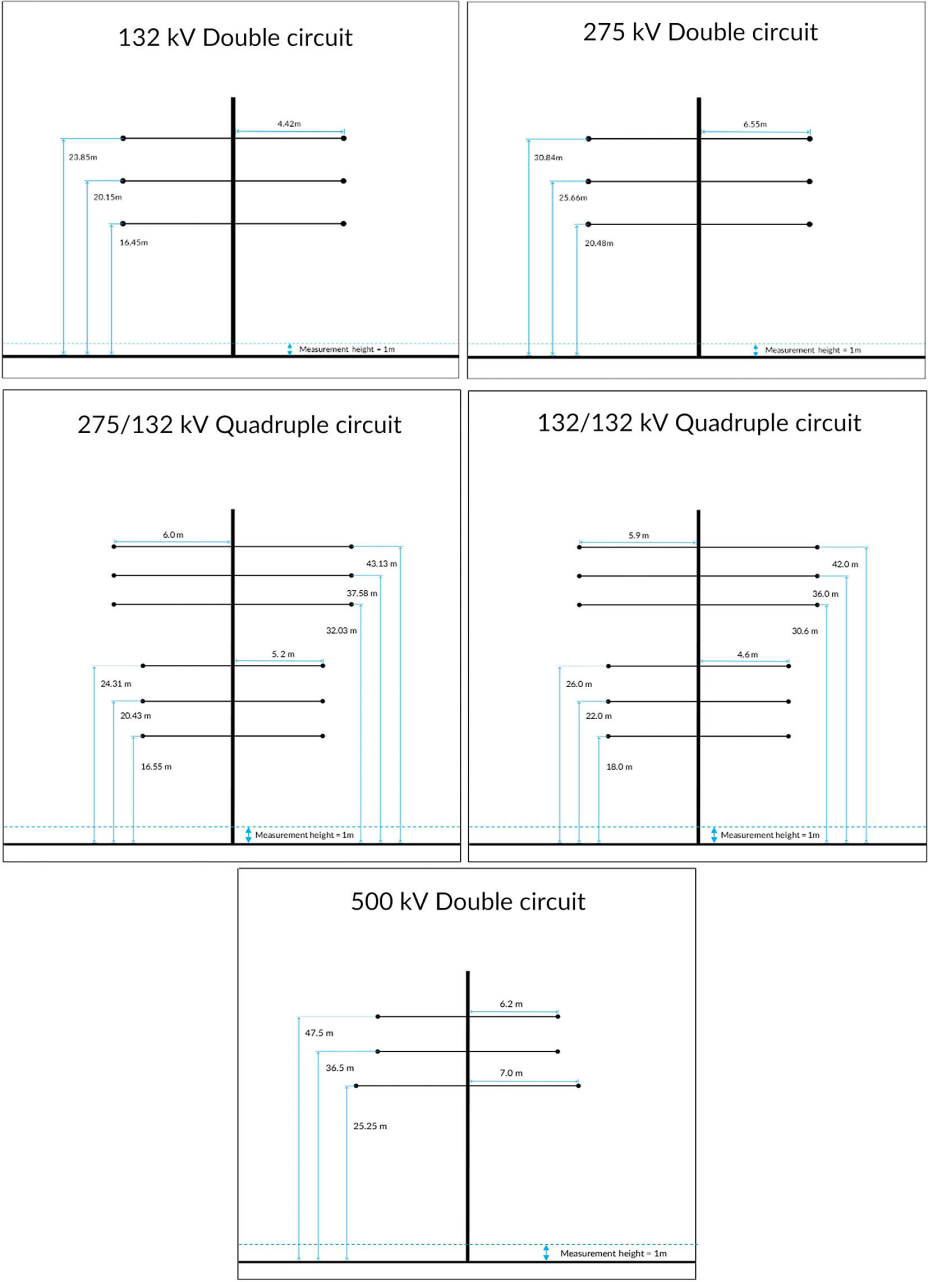
Overhead power transmission lines towers in Malaysia. ^4^^-^^6^^,^^10^^,^^11^

Overhead power transmission lines in Malaysia are typically rated at 132 kV, 275 kV, and 500 kV. Figure 1 shows a typical dimension for various power transmission lines towers in Malaysia. These dimensions agree well with the transmission line design manual in I. Beck. ^4^

Several isolated simulation studies on the propagation of EMF around the 132kV, 275kV, and 500kV power transmission lines in Malaysia has been presented in Sahbudin *et al.,* ^5^ Said and Hussain, ^6^ Jimbin and Ahmad, ^7^ and Rahman *et al.* ^8^ All of the studies referenced here are related to EMF propagation around the power transmission lines and how it affects the nearby public. The studies were conducted in several locations in Malaysia in which the EMF level was analyzed and computed at a specific point and distance from the power transmission line. However, analysis of the compliance of the EMF with recommended public exposure limits remains lacking as no specific study evaluated the EMF level from every type of power transmission line in Malaysia. Hence, in this study, simulations of the electromagnetic field's radiation from various high-voltage power transmission lines in Malaysia are conducted simultaneously to provide an overall comparison in terms of their corresponding EMF level as a function of distance away from the power lines. The simulated EMF levels are then benchmarked with public exposure limits recommended by international standards and the commenced right of way (ROW), after which the minimum safe distance of power transmission lines from residential areas can then be determined.

## Methods

**Figure 2.**
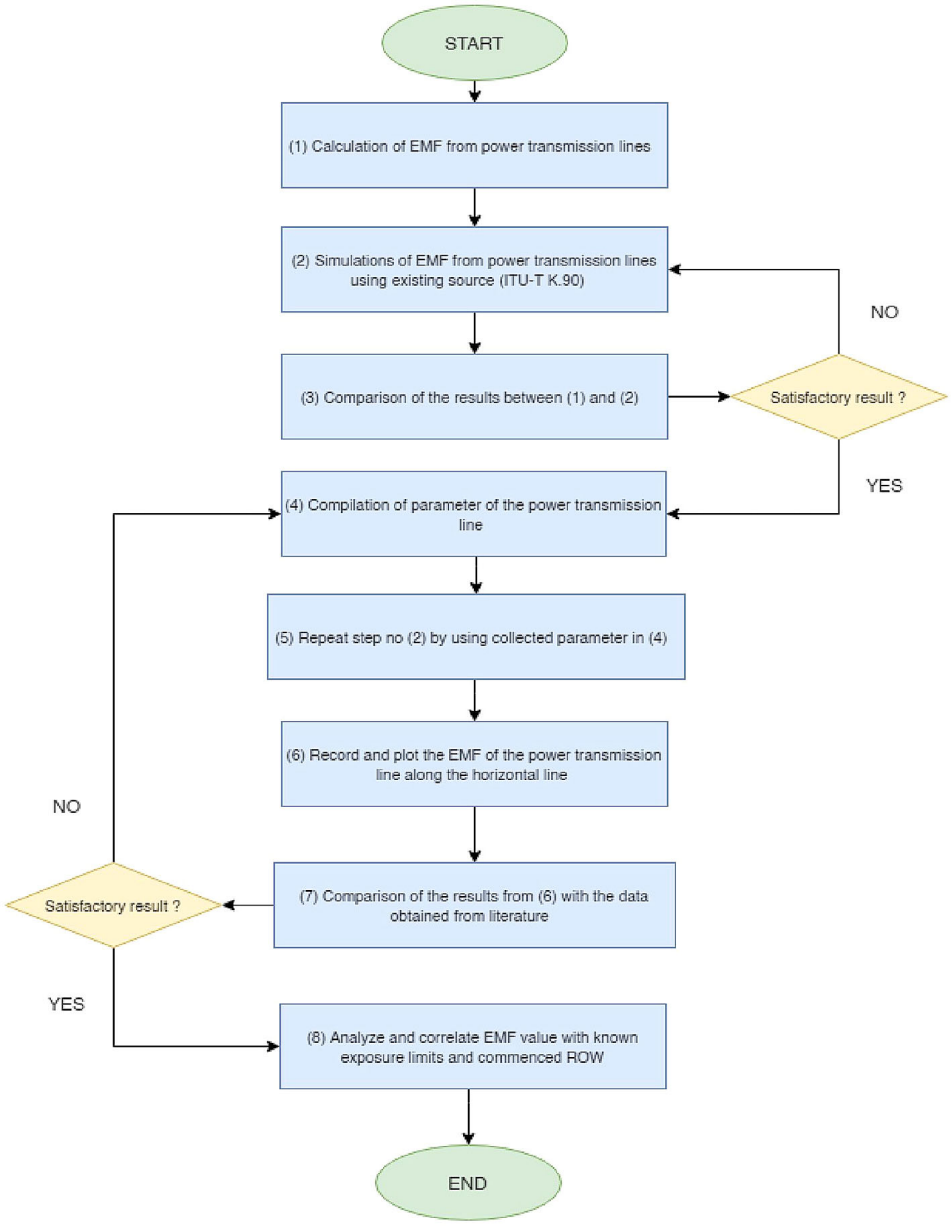
Overall project flow.

**Figure 3.**
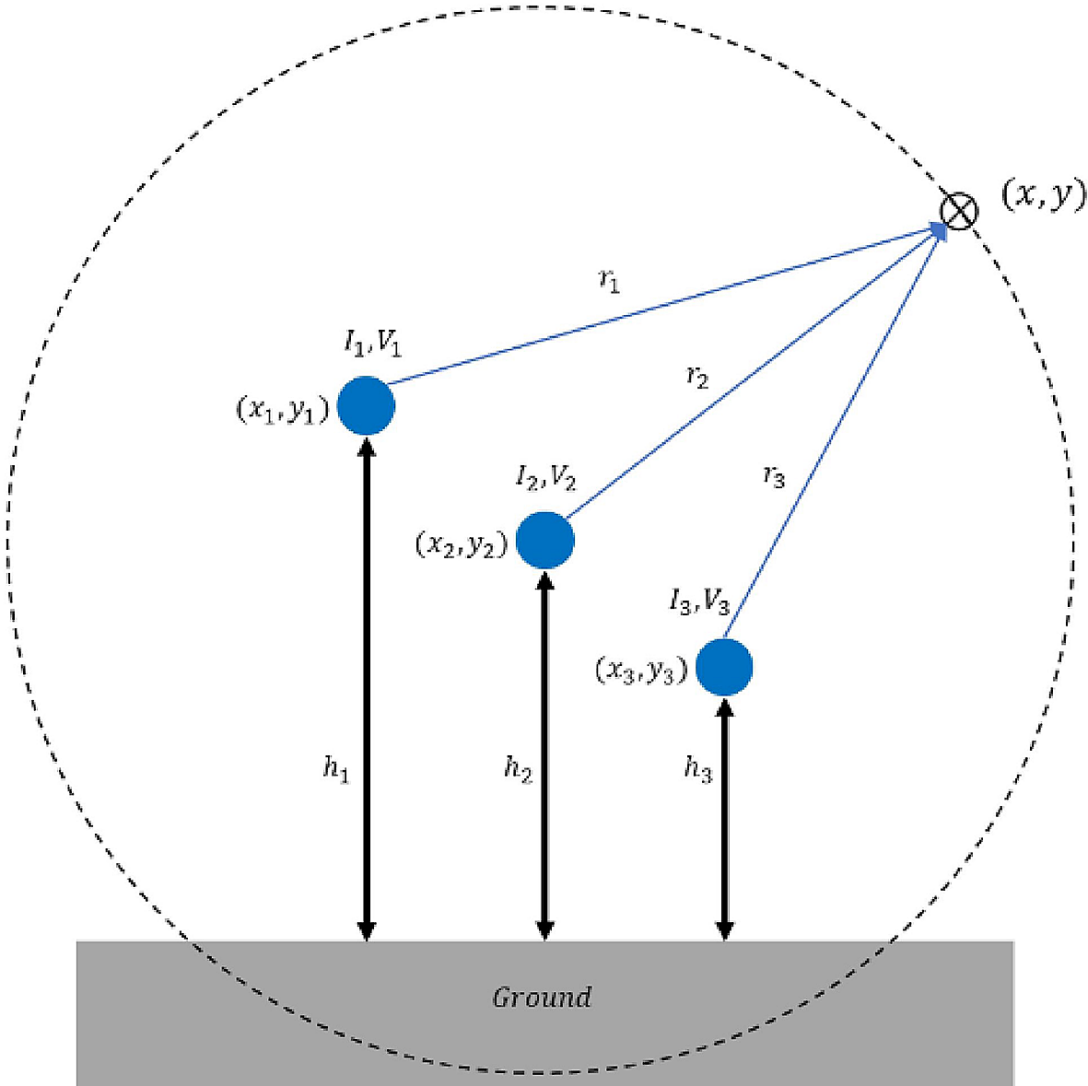
Geometrical illustration for the magnetic and electric field calculation. ^12^

Figure 2 depicts the overall project flow for this work. ^20^ The field calculation for a three-phase circuit power transmission line is based on the principle of superposition. ^9^^,^^12^ Suppose the three conductors are labelled with an index *k* that ranges from one to three. For each conductor, there is a combination of complex current $I_{k}$ and complex voltage $V_{k}$ . The position of the conductors is denoted by $\left(x_{k},y_{k}\right)\;$ in which the origin can be chosen at any point. The objective is to calculate the field at a point of interest ( *x, y*). Every conductor is above the earth at the height of $h_{k}$ and $r_{k}$ away from the point of interest as shown in Figure 3. For a single conductor-carrying current *I* along a straight line, the magnetic field magnitude, *B* is given by:

$$B=\frac{\mu_{o}I_{o}}{2\pi {\left(x^{2}+y^{2}\right)}^{\frac{1}{2}}}$$ (1)

where $\mu_{o}$ : permittivity of free space.

The resultant magnetic field due to a three-phase conductor system is the complex summation of contributing magnetic field vectors. The vertical and horizontal components of *B* can then be established geometrically as shown in the National Grid EMF guide. ^13^ Each conductor is then resolved into an in-phase and out-of-phase component to determine the current relation. This is done by referring to the phasor diagram depicted in the National Grid EMF guide. ^13^ By referring to the phasor diagram, all the in-phase and out-of-phase components can be extracted using the sin and cos of 30° (120° − 90°). For a single three-phase circuit, it does not matter which order these conductors were placed. Each conductor will have a phase 120° apart from the other two conductors. Being 120° apart makes the phases balanced in which the power transfer is constant at any point. This method has also been used in other isolated simulation studies presented in Refs. 6 and 7. For more than one circuit, a consistent convention should be applied when defining the phases to achieve optimum efficiency as shown in the National Grid EMF guide. ^13^

By multiplying both geometrical and current term, four components of the field can be obtained, which is *B*_*in*, *x*_, *B*_*in*, *y*_, *B*_*out*, *x*_, and *B*_*out*, *y*_. The total magnetic field is then given by:

$$B_{\text{total}}=\sqrt{{\left(B_{\text{in},x}\right)}^{2}+{\left(B_{\text{in},y}\right)}^{2}+{\left(B_{\text{out},x}\right)}^{2}+{\left(B_{\text{out},y}\right)}^{2}}$$ (2)

The electric field generated by a system of conductors is attributable to the potential $V_{k}$ applied to each of them. The electric coupling between conductors should be considered as well in order to determine the linear charge density. ^12^ The potential V at its boundary surface for a single conductor of effective radius R' at a height *h* above the ground is given by:

$$V=\frac{\lambda }{2\pi \varepsilon_{0}}\ln\frac{2h}{R\prime }$$ (3)

where λ : equivalent density of charge; $\varepsilon_{0}$ is the permittivity of free space.

The conductor's electrical image's contribution was also incorporated in equation 3 in the case of perfectly conducting ground. For a known value of *V*, the equivalent linear density of charge can be assessed along with its electrical image, which is then used to calculate the electric field vector at any point of interest (x, y). A single contribution to the electric field either from the conductor or its electrical image is given by:

$$\bar{E}=\frac{\lambda }{2\pi \varepsilon_{0}}\frac{1}{r}\hat{r}$$ (4)

where r : distance from the conductor (or image) to the point of interest; $\hat{r}$ is the respective unit vector. In the event of multiple conductors (three conductors for a three-phase circuit), the equivalent linear density of charge is determined by solving the linear system of equations given by:

$$\left[P\right]\left[\lambda \right]=\left[V\right]$$ (5)

where $\;\left[P\right]$ : coupling matrix; $\left[\lambda \right]$ : equivalent densities vector; $\left[V\right]\;$ : vector of potentials applied to the conductors.

The coupling matrix can be defined as:

$$P_{\mathrm{ii}}=\frac{1}{2\pi \varepsilon_{0}}\ln\frac{2h_{i}}{R_{i}}\;P_{\mathrm{ij}}=\frac{1}{2\pi \varepsilon_{0}}\ln\frac{D_{\mathrm{ij}}}{d_{\mathrm{ij}}}$$ (6)

Here the conductor indexes are denoted by *i j* from 1 to *N.* $D_{\mathrm{ij}}$ represents the distance between the conductor *i* and the image of the conductor *j* while $d_{\mathrm{ij}}$ represents the distance between the conductor *i* and the conductor *j.* With *V* as the voltage level of the overhead power transmission lines in Malaysia as depicted in Figure 1, the charge density can be obtained by inverting the matrix equation above where:

$$\left[\lambda \right]=\left[V\right]\;{\left[P\right]}^{-1}$$ (7)

After solving the system of equations, the corresponding charge densities are then used to calculate the resultant electric field at the point of interest, as in the case of a single conductor described above. The resulting electric field due to a system of conductors (and their electric images) is obtained by the complex summation of the contributing electric field vectors.

In this study, the EMF simulations were conducted using the publicly available EMFACDC v2.0, update to Appendix II program developed by the Telecommunication Standardization Sector of International Telecommunication Union (ITU-T) under the series of recommendations known as Series K ( https://www.itu.int/rec/T-REC-K.90-201905-I!Amd1/en). Table 1 shows the geometrical parameters of this study.

**Table 1.** Geometrical parameters.

Coordinates of plotted area				
x1	x2	y1	y2	Step
−30 m	30 m	0 m	40 m	0.1 m

Parameters for the line chart	
Horizontal line, Y–coordinate	Vertical line, X–coordinate
1 m (in compliance with *IEEE Std C37.1* ^14^)	0 m

Verification of the EMFACDC program is performed by comparing the simulation value obtained from the program with the value obtained through manual calculation performed for a typical power line setup. To do this, the exact parameter of the selected power line is keyed into the program. The results were considered satisfactory if both of the values were approximately similar to each other. This study chooses the magnetic field level of a typical United Kingdom (UK) 400kV double circuit power transmission line at 10 meters from the centerline. As this verification purpose is to test for the accuracy of the simulation program, any type of power line is applicable.

**Figure 4.**
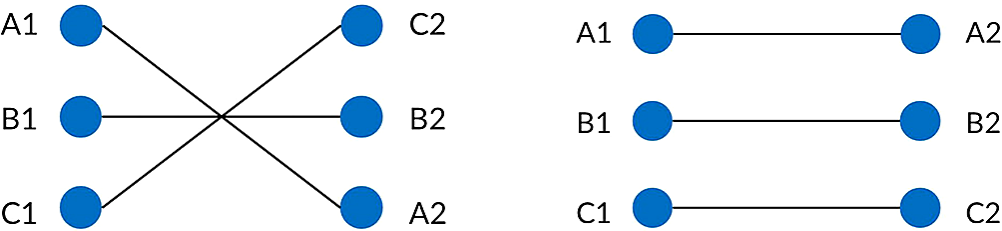
Double circuit phase configuration for standard transposed phasing (left) and un-transposed phasing (right).

**Figure 5.**
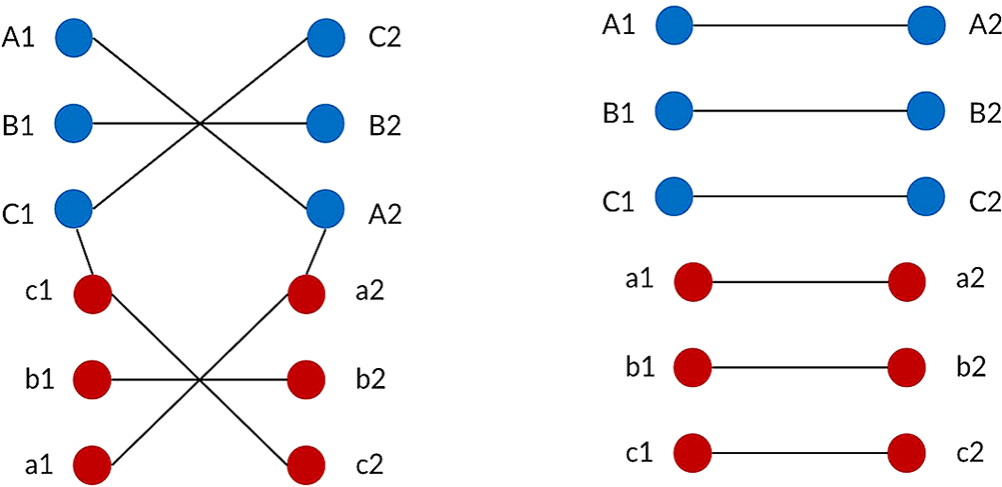
Quadruple circuit phase configuration for standard transposed phasing (left) and un-transposed phasing (right).

Rated current is used for each conductor in the power lines to test for the maximum current value possible as applied in Jimbin and Ahmad. ^7^ The current in the left and right circuits are assumed to be equal in order to ensure the EMF level is balanced at the left and right ROW boundary. For each type of power transmission line, the conductors are represented by their phase pattern and assigned their own phase code. Standard transposed phasing and un-transposed phasing will be considered in this study as shown in Figures 4 and 5. Transposition of phasing is performed to reduce crosstalk or interference which arises by current flowing in the conductors. Therefore, un-transposed phasing is expected to produce the highest magnetic flux density and vice versa, as suggested in Said *et al.,* ^6^ under Group 5 conductor phase pattern. Although transposed phasing is used by most of the power transmission lines in the National Grid to significantly reduce the magnetic and electric field generated, un-transposed phasing is still considered in this study as it is not always feasible to transpose every line.

The parameters for each type of power lines which are based on Malaysia’s specific power transmission line were consolidated from R. K. Z. Sahbudin *et al.,* ^5^ I. Said *et al.,* ^6^ V. S. Jimbin and Ahmad, ^7^ N. A. Rahman *et al.,* ^8^ I. M. Rawi *et al.,* ^10^ and N. H. N. Hassan *et al.* ^11^ These dimensions also agree well with the Transmission Line Design Manual prepared by R. W. Beck, Inc., for the use of Tenaga Nasional Berhad (TNB) in June 2000. ^4^ Five types of power transmission lines available in Malaysia are considered in this study. These are the power transmission lines typically used in Malaysia, known with voltage levels of 132 kV, 275 kV, and 500 kV:

1. 132 kV double circuit power transmission line.
2. 275 kV double circuit power transmission line.
3. 132/132 kV quadruple circuit power transmission line.
4. 275/132 kV quadruple circuit power transmission line.
5. 500 kV double circuit power transmission line.

These power transmission lines were identified based on the Suruhanjaya Tenaga Wayleave for Electricity Supply Lines. ^15^ They were chosen as they were categorized under high voltage and extra-high voltage distribution and ran under three-phase configurations. Tables 2 and 3 show samples of the input data used for the field simulation of 275/132kV quadruple circuit power line and 500 kV double circuit power lines in Malaysia. ^7^^,^^8^^,^^10^ The remaining set of input data i.e. of 132 kV double circuit power line, 275 kV double circuit power line and 132/132 kV quadruple circuit power line is made available in Tables 3, 4, and 5. ^20^

**Table 2.** 275/132 kV quadruple circuit power line.

No	Phase code		Cond. coordinate		Current rating (A)	Voltage level (kV)	Phase angle (Deg.)	
	U	T	x(m)	y(m)			U	T
1	A1	A1	−6.0	43.13	1232	275	0	0
2	B1	B1	−6.0	37.58	1232	275	120	120
3	C1	C1	−6.0	32.03	1232	275	240	240
4	A2	C2	6.0	43.13	1232	275	0	240
5	B2	B2	6.0	37.58	1232	275	120	120
6	C2	A2	6.0	32.03	1232	275	240	0
7	a1	c1	−5.2	24.31	729	132	0	240
8	b1	b1	−5.2	20.43	729	132	120	120
9	c1	a1	−5.2	16.55	729	132	240	0
10	a2	a2	5.2	24.31	729	132	0	0
11	b2	b2	5.2	20.43	729	132	120	120
12	c2	c2	5.2	16.55	729	132	240	240

**Table 3.** 500 kV double circuit power line.

No	Phase code		Cond. coordinate		Current rating (A)	Voltage level (kV)	Phase angle (Deg.)	
	U	T	x(m)	y(m)			U	T
1	A1	A1	−6.2	47.5	2309.4	500	0	0
2	B1	B1	−6.2	36.5	2309.4	500	120	120
3	C1	C1	−7.0	25.25	2309.4	500	240	240
4	A2	C2	6.2	47.5	2309.4	500	0	240
5	B2	B2	6.2	36.5	2309.4	500	120	120
6	C2	A2	7.0	25.25	2309.4	500	240	0

**Table 4.** Benchmarked public exposure limits.

No	Category	Public exposure limit
**1.**	Specific International Organization	ICNIRP 2010 ^16^ ( **5 kV/m** for electric field and **200 μT** for magnetic field)
**2.**	National Standardization Committee	EU Recommendation 1999 ^17^ ( **5 kV/m** for electric field and **100 μT** for magnetic field)
**3.**	Regional variation of a country	Exposure limit for the residential region – Slovenia and Italy ^18^ ( **0.5 kV/m** for electric field and **10 μT** for magnetic field)

**Table 5.** Magnetic field of the power transmission lines in Malaysia.

Type of power lines	Conducting ground		Phasing		Magnetic flux densities (μT)		Compliance to exposure limit?		
	Yes	No	Untransposed	Transposed	Under the line	ROW boundary	ICNIRP	EU	Slovenia/Italy
132 kV double circuit	✓		✓		4.794284	0.253069	Yes	Yes	Yes
	✓			✓	0.545671	0.624946	Yes	Yes	Yes
		✓	✓		2.6043	1.4302	Yes	Yes	Yes
		✓		✓	1.3384	0.4673	Yes	Yes	Yes
275 kV double circuit	✓		✓		3.928109	0.960835	Yes	Yes	Yes
	✓			✓	0.443016	1.074408	Yes	Yes	Yes
		✓	✓		2.088845	1.494544	Yes	Yes	Yes
		✓		✓	1.263348	0.636509	Yes	Yes	Yes
132/132 kV quadruple circuit	✓		✓		13.62006	2.978469	Yes	Yes	Yes
	✓			✓	1.13004	0.340224	Yes	Yes	Yes
		✓	✓		7.28901	4.670191	Yes	Yes	Yes
		✓		✓	1.493262	0.676831	Yes	Yes	Yes
275/132 kV quadruple circuit	✓		✓		10.13539	2.489524	Yes	Yes	Yes
	✓			✓	0.909531	0.18436	Yes	Yes	Yes
		✓	✓		5.405135	3.546732	Yes	Yes	Yes
		✓		✓	1.113876	0.588538	Yes	Yes	Yes
500 kV double circuit	✓		✓		18.43185	4.860459	Yes	Yes	Yes
	✓			✓	2.857379	4.293753	Yes	Yes	Yes
		✓	✓		9.693522	6.762919	Yes	Yes	Yes
		✓		✓	4.852491	2.516856	Yes	Yes	Yes

The simulation of electric and magnetic fields at various horizontal distance from the center of power lines was analyzed and benchmarked with public exposure limits from selected organizations defined in Table 4 (Table 8 ^20^). The International Commission on Non-Ionizing Radiation Protection (ICNIRP) and European Union (EU) limits were selected as they were used in most of the countries globally while Slovenia and Italy were purposely selected due to their more stringent requirement. In this study, public exposure limits from three different categories are chosen for the correlation studies to satisfy all variants in every country. The exposure limits selected were used to prevent established effects at high electromagnetic field level. The simulation is also benchmarked with the commenced ROW from Suruhanjaya Tenaga for each type of power lines to estimate the electromagnetic field level at the minimum separation distance from the center of power lines. ^15^ The ROW specified by the Suruhanjaya Tenaga 15 is applicable for the correlation process as it is the currently commenced minimum safe distance for power transmission lines in Malaysia. Correlation between the exposure limit and the commenced ROW is analyzed by observing the electromagnetic field level at the minimum separation distance. The field level at the minimum separation distance is considered to be safe if it is lower than the exposure limits.

## Results

The simulation was initially verified with a typical setup of a double circuit power transmission line in the United Kingdom ^19^ as outlined in the methods section (Table 2 ^20^). Upon verification, simulation of magnetic flux densities and the electric field strength from different types of power lines at the horizontal line were conducted. The magnetic and electric field of the power transmission lines relative to the selected exposure limits and commenced ROW were summarized and shown in Tables 5 and 6 (Table 10 and Table 11 ^20^).

**Table 6.** Electric field of the power transmission lines in Malaysia.

Type of power lines	Conducting ground		Phasing		Electric field strength (kV/m)		Compliance to exposure limit?		
	Yes	No	Untransposed	Transposed	Under the line	ROW boundary	ICNIRP	EU	Slovenia/Italy
132 kV double circuit	✓		✓		0.924003	0.24556	Yes	Yes	Yes
	✓			✓	0.15441	0.17748	Yes	Yes	Yes
		✓	✓		0.263679	0.17961	Yes	Yes	Yes
		✓		✓	0.182788	0.121504	Yes	Yes	Yes
275 kV double circuit	✓		✓		1.50842	0.574706	Yes	Yes	No
	✓			✓	0.222091	0.450741	Yes	Yes	Yes
		✓	✓		0.394063	0.305229	Yes	Yes	Yes
		✓		✓	0.276856	0.181895	Yes	Yes	Yes
132/132 kV quadruple circuit	✓		✓		1.12788	0.367053	Yes	Yes	Yes
	✓			✓	0.354182	0.319246	Yes	Yes	Yes
		✓	✓		0.321052	0.211805	Yes	Yes	Yes
		✓		✓	0.105488	0.081465	Yes	Yes	Yes
275/132 kV quadruple circuit	✓		✓		1.48149	0.57809	Yes	Yes	No
	✓			✓	0.534335	0.450279	Yes	Yes	Yes
		✓	✓		0.457699	0.31446	Yes	Yes	Yes
		✓		✓	0.135271	0.113173	Yes	Yes	Yes
500 kV double circuit	✓		✓		3.08961	1.09486	Yes	Yes	No
	✓			✓	0.651354	0.74896	Yes	Yes	No
		✓	✓		0.724198	0.539665	Yes	Yes	No
		✓		✓	0.414757	0.232802	Yes	Yes	Yes

The magnetic field due to all the power transmission lines are within the exposure limits set by the organizations in Table 4 (Table 8 ^20^). The highest magnetic flux densities at the ROW boundary were simulated from the un-transposed 500kV double circuit power line under non-conducting ground condition as shown in Table 10. ^20^ Even directly below the overhead power lines, the magnetic field levels are still within the exposure limit recommended by ICNIRP and EU. ^16^^,^^17^

Similarly, the electric field due to all the power transmission lines are within with the exposure limits set by ICNIRP and EU. However, 25% of the power transmission lines exceed the stricter exposure limit practiced in Slovenia and Italy. The highest electric field strength data at the ROW boundary was simulated from the 500kV double circuit power line under un-transposed and conducting ground condition (Table 11 ^20^). Similar to the magnetic field simulation, even below the overhead power lines, the electric field levels are still within the exposure limit recommended by ICNIRP 2010 and EU Recommendation 1999. ^16^^,^^17^ The transposed conductors give significantly lower magnetic flux densities and electric field strength.

## Conclusions

The electric and magnetic field due to five types of power transmission lines in Malaysia were simulated and benchmarked with the known exposure limits and commenced ROW. The electric and magnetic field level for all types of power lines at the edge of ROW was found to be lower than the exposure limit recommended by ICNIRP. Hence, it can be concluded that Malaysia's power transmission lines are within the safe exposure limit recommended by ICNIRP. To further minimize the electric and magnetic field level, it is recommended that the residential building should be built at least 30 meters away from the power transmission lines, especially for the 275kV double circuit, 275/132kV quadruple circuit, and 500kV double circuit lines.

## Software availability

The software EMFACDC v2.0, appendix 1 (2020) which is used in simulating the electric and magnetic field in this study is publicly available to download at https://www.itu.int/rec/T-REC-K.90-201905-I!Amd1/en.

## Author roles

Azhan F.: Conceptualization, Formal Analysis, Methodology, Writing – Original Draft Preparation, Writing – Review & Editing;

Siow C.L.: Conceptualization, Supervision, Writing – Review & Editing

M. Zainal A.A.K.: Conceptualization, Writing – Review & Editing

## Data availability statement

### Underlying data

Figshare: Data for Electric and Magnetic Field of Power Transmission Lines in Malaysia.

https://doi.org/10.6084/m9.figshare.16577423.v2. ^20^

The project contains the following underlying data:

• Data for Electric and Magnetic Field of Power Transmission Lines in Malaysia. (Simulated dataset of electric and magnetic field of five types of power transmission lines in Malaysia in Tables 1-12 and Figures 1-20).
• Figure 1. (Overhead power transmission lines towers in Malaysia).
• Figure 2. (Overall project flow).
• Figure 3. (Geometrical illustration for the magnetic and electric field calculation).
• Figure 4. (Double circuit phase configuration for standard transposed phasing (left) and un-transposed phasing (right).
• Figure 5. (Quadruple circuit phase configuration for standard transposed phasing (left) and un-transposed phasing (right).

Data are available under the terms of the Creative Commons Zero “No rights reserved” data waiver (CC0 1.0 Public domain dedication).
