# Supplementary material for: EMI radiation of power transmission lines in Malaysia
Source: F1000Res. 2022 Feb 24;10:1136. Originally published 2021 Nov 10. [Version 2] doi: 10.12688/f1000research.73067.2 (PMC8941290; doi:10.12688/f1000research.73067.2)
Supplement: Peer review history

# Peer review: Reviewer response for version 2

**DOI:** 10.5256/f1000research.121539.r125173

**Published:** 2022-03-22

**Authors:** Muhammad Ammirrul Atiqi Mohd Zainuri

**Affiliations:** 1 Electrical, Electronic and Systems Engineering, Faculty Engineering and Built Environment, Universiti Kebangsaan Malaysia, Bangi, Malaysia

Well-written manuscripts. It is advised to double-check back any grammatical errors throughout the manuscripts. The discussion needs to be added more especially in referring to table 6.

Is the work clearly and accurately presented and does it cite the current literature?

Yes

If applicable, is the statistical analysis and its interpretation appropriate?

Yes

Are all the source data underlying the results available to ensure full reproducibility?

Yes

Is the study design appropriate and is the work technically sound?

Yes

Are the conclusions drawn adequately supported by the results?

Yes

Are sufficient details of methods and analysis provided to allow replication by others?

Yes

Reviewer Expertise:

Power Electronics, Power Quality, Electrical Power Engineering, Renewable Energy System, Artificial Intelligence and Agriculture-Photovoltaic.

I confirm that I have read this submission and believe that I have an appropriate level of expertise to confirm that it is of an acceptable scientific standard.

# Peer review: Reviewer response for version 2

**DOI:** 10.5256/f1000research.121539.r125172

**Published:** 2022-02-25

**Authors:** Jacek Paś

**Affiliations:** 1 Division of Electronic Systems Exploitations, Faculty of Electronics, Institute of Electronic Systems, Military University of Technology, Warsaw, Poland

I have studied to improve and revise the entire article again for the comments I made. 

 The authors have incorporated all criticisms into the revised article.

 The development of the article by the authors after taking into account the comments is good. The article can be accepted for indexing.

 The response to the reviewer's comments by the authors is very good. The authors can further extend the research on the environmental impact of high voltage lines.

Is the work clearly and accurately presented and does it cite the current literature?

Yes

If applicable, is the statistical analysis and its interpretation appropriate?

I cannot comment. A qualified statistician is required.

Are all the source data underlying the results available to ensure full reproducibility?

Yes

Is the study design appropriate and is the work technically sound?

Yes

Are the conclusions drawn adequately supported by the results?

Yes

Are sufficient details of methods and analysis provided to allow replication by others?

Partly

Reviewer Expertise:

NA

I confirm that I have read this submission and believe that I have an appropriate level of expertise to confirm that it is of an acceptable scientific standard.

# Peer review: Reviewer response for version 1

**DOI:** 10.5256/f1000research.76690.r99796

**Published:** 2022-02-07

**Authors:** Muhammad Ammirrul Atiqi Mohd Zainuri

**Affiliations:** 1 Electrical, Electronic and Systems Engineering, Faculty Engineering and Built Environment, Universiti Kebangsaan Malaysia, Bangi, Malaysia

Introduction:

- Add proper objectives like "this paper or manuscript present... "
- Add also an overview of each section at the end of the introduction part. 

Methods: 

- Explain in more detail the flowchart of Figure 2.

Results:

- Do you have any waveform that can show the EMI related to Electric field strength?
- More discussion needed as referred to Table 6 in reference to each parameter discussed as in table 6. 

Conclusions:

- Too short, add more summary on the methods and results. 

Is the work clearly and accurately presented and does it cite the current literature?

Yes

If applicable, is the statistical analysis and its interpretation appropriate?

Yes

Are all the source data underlying the results available to ensure full reproducibility?

Yes

Is the study design appropriate and is the work technically sound?

Yes

Are the conclusions drawn adequately supported by the results?

Yes

Are sufficient details of methods and analysis provided to allow replication by others?

Yes

Reviewer Expertise:

Power Electronics, Power Quality, Electrical Power Engineering, Renewable Energy System, Artificial Intelligence and Agriculture-Photovoltaic.

I confirm that I have read this submission and believe that I have an appropriate level of expertise to confirm that it is of an acceptable scientific standard, however I have significant reservations, as outlined above.

# Response

**Published:** 2022-02-20

**Authors:** Chun Lim Siow

**Affiliations:** Multimedia University, Malaysia

Thank you for the invaluable comments to improve this paper. The improvements made are summarised below:

 Introduction:

- Add proper objectives like "this paper or manuscript present... "
* The objective has been rephrased as follows:*
*Hence, this paper presents the simulations of the electromagnetic field radiation from various high-voltage power transmission lines in Malaysia conducted simultaneously to provide an overall comparison in terms of their corresponding EMF level as a function of distance away from the power lines.*
- Add also an overview of each section at the end of the introduction part.
*The overview of each section has been added at the end of the introduction part.* 

Methods: 

- Explain in more detail the flowchart of Figure 2.
*Flowchart of Figure 2 has been explained in more detail under the Methods section.*

Results:

- Do you have any waveform that can show the EMI related to Electric field strength? 
*An additional Reference 21 has been cited to support the relation between EMI and electric field strength. *
- More discussion needed as referred to Table 6 in reference to each parameter discussed as in table 6. 
*The discussion on Table 6 has been extended*

Conclusions:

- Too short, add more summary on the methods and results. 
*The conclusion has been expanded to be more comprehensive.*

# Peer review: Reviewer response for version 1

**DOI:** 10.5256/f1000research.76690.r120587

**Published:** 2022-02-07

**Authors:** Jacek Paś

**Affiliations:** 1 Division of Electronic Systems Exploitations, Faculty of Electronics, Institute of Electronic Systems, Military University of Technology, Warsaw, Poland

1. Please add at the end of the article a list of abbreviations used in the article.
2. The critical review of the literature on the state of the issue is done poorly.
3. Please expand the conclusions in the article, they are currently only four sentences long. Not all important graphs are included in the conclusions.
4. The article and low frequency electromagnetic field calculations around power lines are very interesting, and the results can be used to determine safety zones.
5. The issue of low-frequency electromagnetic field effects is important to the health of people living near power lines. Please refer to the E, H component limits of standards in other countries, e.g. USA, European countries and the proposed regulations in the country.

Is the work clearly and accurately presented and does it cite the current literature?

Yes

If applicable, is the statistical analysis and its interpretation appropriate?

I cannot comment. A qualified statistician is required.

Are all the source data underlying the results available to ensure full reproducibility?

Yes

Is the study design appropriate and is the work technically sound?

Yes

Are the conclusions drawn adequately supported by the results?

Yes

Are sufficient details of methods and analysis provided to allow replication by others?

Partly

Reviewer Expertise:

Electronic safety systems, compatibility

I confirm that I have read this submission and believe that I have an appropriate level of expertise to confirm that it is of an acceptable scientific standard, however I have significant reservations, as outlined above.

# Response

**Published:** 2022-02-20

**Authors:** Chun Lim Siow

**Affiliations:** Multimedia University, Malaysia

Thank you for your invaluable comments to improve this article. The improvements made are summarised below:

1. Please add at the end of the article a list of abbreviations used in the article.
   
*The list of abbreviations has been added at the end of the article.*
2. The critical review of the literature on the state of the issue is done poorly.
* The literature review has been improved. The contributions from related and cited articles have been discussed and the research gap has been clearly identified and stated.*
3. Please expand the conclusions in the article, they are currently only four sentences long. Not all important graphs are included in the conclusions.
   
*The conclusion has been expanded to be more comprehensive.*
4. The article and low frequency electromagnetic field calculations around power lines are very interesting, and the results can be used to determine safety zones.
   
*Yes, this is indeed an important issue where public awareness and understanding still has a lot of room for improvement.*
5. The issue of low-frequency electromagnetic field effects is important to the health of people living near power lines. Please refer to the E, H component limits of standards in other countries, e.g. USA, European countries and the proposed regulations in the country.
*Thank you for the recommendation. The low frequency electromagnetic field effect is indeed a very important issue for the health of people living near power lines. In this paper, we have benchmarked against the world’s largest and most relevant organization namely ICNIRP, which is collaborating with many other relevant stakeholders such as World Health Organisation and national radiation protection bodies at the global level. We have also benchmarked against a regional recommendation which is European Union and two European countries (Slovenia and Italy) which sets more stringent exposure limits for the E and H components. For future work, it would be interesting to consider USA (with so many large states) and other European countries as per your recommendation. *
